# Meta-Analysis of the Relationship between NM23 Expression to Gastric Cancer Risk and Clinical Features

**DOI:** 10.1155/2017/8047183

**Published:** 2017-03-16

**Authors:** Min Fang, Yifeng Tao, Zhimin Liu, Hao Huang, Min Lao, Lingsha Huang, Bo Zhu

**Affiliations:** Department of Clinical Laboratory, The Affiliated Tumor Hospital of Guangxi Medical University, Nanning, Guangxi, China

## Abstract

The prognostic value of reduced NM23 expression for gastric cancer (GC) patients is still contradictory. Thus, we conducted a meta-analysis to quantitatively evaluate the association of NM23 expression with GC risk and clinical features by analyzing 27 publications. The result of our meta-analysis indicated that NM23 expression is markedly reduced in gastric cancer tissues (OR = 3.15; 95% CI = 1.97–5.03; *P* < 0.001). Furthermore, NM23 expression was negatively correlated with N stage, TNM stage, and histological grade. However, NM23 expression was not correlated with T stage, lymphatic invasion, vascular invasion, and 5-year overall survival rate. In conclusion, reduced NM23 expression correlated with gastric cancer risk, but its association with GC clinical features remains inconclusive. Therefore, large-scale and well-designed studies, which use uniform antibody and criterion of NM23 positive expression, are required to further validate the role of the NM23 in predicting GC progression.

## 1. Introduction

Gastric cancer (GC) is one of the most common gastrointestinal cancers and the second leading cause of cancer related death worldwide [1]. The incidence and mortality rate is especially high in Eastern Asia populations including China, Japan and Korea, which may be on account of a high prevalence of chronic* Helicobacter pylori* infection, diets rich in salt, and genetics background [2]. Although the clinical prognosis for GC has been improved by the development of early detection and adjuvant chemoradiotherapy, the 5-year overall survival (OS) rate for GC patients worldwide is still less than 25% [3]. Therefore, it is crucial to identify prognostic biomarker for GC and provide clinical treatment strategies to GC patients. Several previous reports showed that reduced expression of nonmetastatic protein 23 (NM23) correlates with tumor pathology and GC disease prognosis [4–6].

NM23 was initially found in metastatic cell lines by Steeg et al. in 1988 and was the first of what has become a field of over 20 known metastasis suppressor genes [7, 8]. In humans, there are 10 genes belonging to the NM23 gene family, of which the two most abundantly expressed are NM23-H1 and NM23-H2 that encode the A and B subunits of nucleoside diphosphate kinase, respectively [9]. The NM23 are involved in multiple-biological processes, such as cellular proliferation, differentiation, motility, and tumor metastasis [10]. Many studies exhibited that reduced expression of NM23 has been regarded as an indicator closely related to the metastasis of tumors, such as hepatocellular, gastric, and colon carcinoma [4, 11, 12]. In contrast, overexpression of NM23 is related to tumors, such as neuroblastoma [13], lymphoma [14], and lung tumor [15], suggesting that the significance of NM23 expression is different depending on cancer types and the NM23 isoforms. As for gastric cancer, numerous studies concerning the NM23 and GC have been performed; however, the results remain controversial. Chen et al. found that the NM23 protein expression was significantly related to lymph node and peritoneal metastasis [5]. On the other hand, some studies showed opposite results or no significant findings. Dhar et al. demonstrated that the overexpression of NM23 in the primary tumors correlated with tumor invasion, metastasis, and progression [16]. Radovic et al. found that NM23 protein expression did not correlate with lymphovascular invasion and lymph node metastases [17]. As the cited articles referred to the analysis were not distinguished NM23 isoforms, we performed a meta-analysis to evaluate whether reduced total NM23 expression is a risk factor for GC and determine its importance as a predictor of disease progression and prognosis of GC. To the best of our knowledge, this is the first comprehensive meta-analysis exploring the prognostic role of NM23 in GC patients.

## 2. Materials and Methods

### 2.1. Literature Search

We collected a systematic literature search from PubMed, Web of Science, and Embase database up to June 20, 2016. The following search terms were used: “gastric cancer”, “gastric carcinoma” or “stomach neoplasms” and “NM23”. We further performed a manual search to identify additional relevant papers.

### 2.2. Study Selection

Published articles were enrolled in our meta-analysis if they conformed to the inclusion criteria: (1) clear data were presented and related to an association between NM23 expression, clinical characteristics, and 5-year OS rate of GC patients; (2) the studies contained adequate published data; (3) it was published as a full-text article restricted to English or Chinese.

### 2.3. Data Extraction

All data from the eligible studies were extracted by two independent investigators with a predefined table. For every eligible study, information collected included the following: the first author's name, publication year, original country, TNM stage of patients, methods for detecting NM23, and 5-year OS rate. We first estimated the association between NM23 expression and GC risk. Moreover, we mainly examined the association between NM23 expression and clinical factors, including T stage, N stage, histological grade, lymphatic invasion, vascular invasion, TNM stage, and 5-year OS rate.

### 2.4. Statistical Analysis

The strength of the association between the NM23 expression and GC risk or clinical factors were estimated by OR with the corresponding 95% CI. In the course of data pooling, statistical heterogeneity was evaluated by using *Q*-test and *I*^2^ test [18]. A *P* value < 0.10 and/or *I*^2^ > 50% are considered significant heterogeneity, and then a random-effect model is employed. Otherwise, a fixed-effect model is used. Furthermore, we assessed publication bias with funnel plot and Egger's linear regression test [19]. *P* value ⩽ 0.05 was considered statistically significant. Statistical analyses were performed with STATA software version 12.0.

## 3. Results

### 3.1. Study Selection and Characteristics

**Figure 1:**
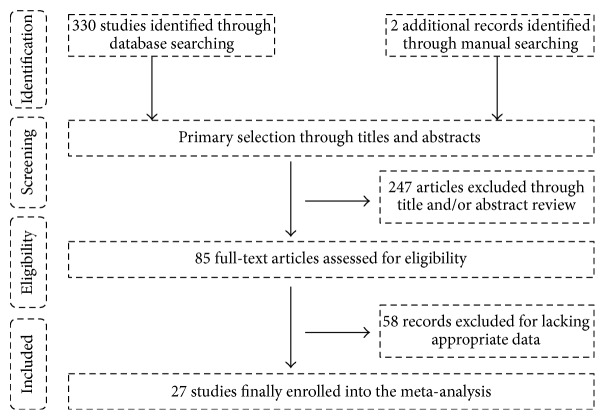
The flowchart of the study selection process.

Systematic database literature search and complementary manual search retrieved a total of 332 relevant articles. After the titles and abstracts were scanned, 247 of the articles were excluded because they were duplicates and unrelated to the research topic. Through reading the remaining articles, 58 studies were excluded because they were nonhuman experiments and nonoriginal full articles and did not provide the appropriate data. Eventually, a total of 27 cohort studies [4–6, 16, 17, 20–41] were enrolled into the analysis. The flow diagram of the study selection process was shown in Figure 1 and the main characters of the 27 studies were summarized in Table 1.

### 3.2. Reduced NM23 Expression Correlated with Gastric Cancer Risk

**Figure 2:**
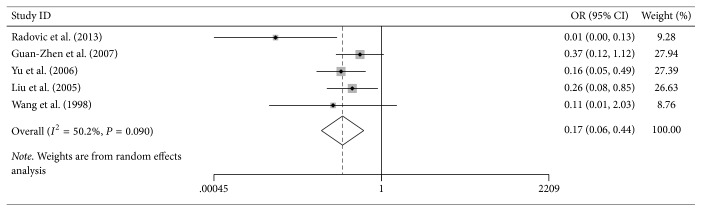
Association of NM23 expression with gastric cancer risk.

There were 5 studies evaluating NM23 expression with GC risk. The results of correlation between NM23 expression and the risk of GC were shown in Figure 2. In general, our study indicated that NM23 expression was statistically significantly declined in gastric cancer patients compared with noncancer controls (OR = 3.15, 95% CI = 1.97–5.03, *P* < 0.001). It is worth mentioning that the degree of heterogeneity was apparent among these studies.

### 3.3. Association between Reduced NM23 Expression and Clinical Features of GC Patient

**Figure 3:**
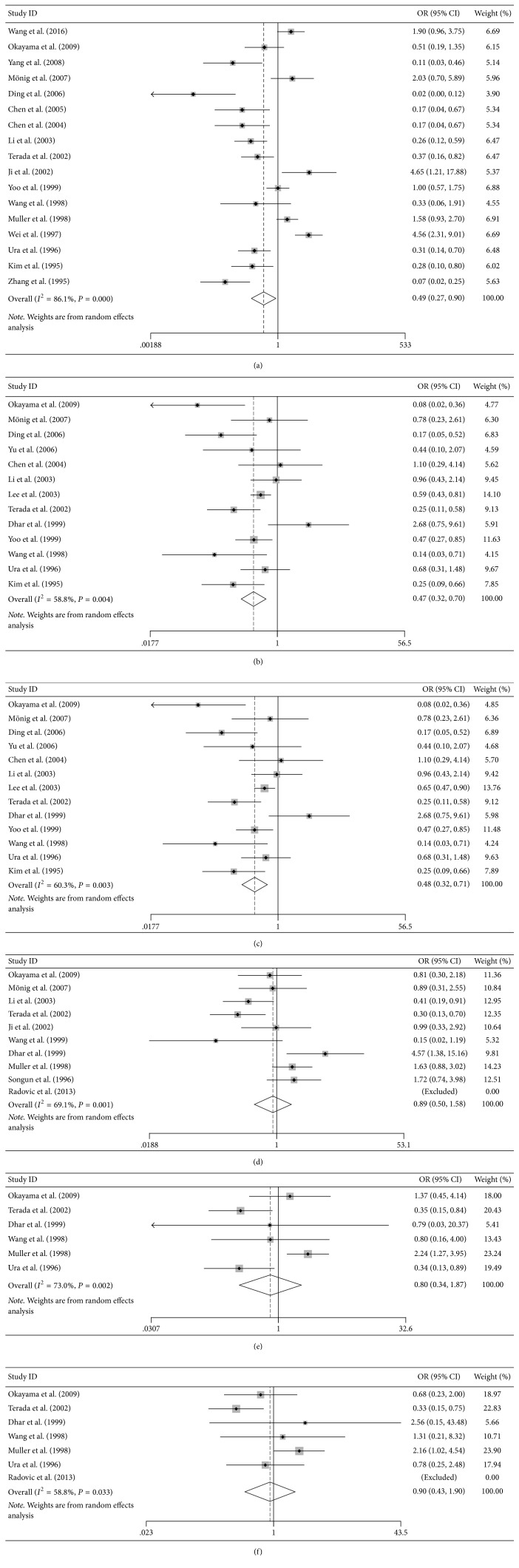
Association of NM23 expression with clinical features. (a) N stage; (b) TNM stage; (c) histological grade (d) T stage; (e) lymphatic invasion; (f) vascular invasion. Reduced NM23 expression was associated with TNM stage and histological grade. However, no significant correlation was found with the other clinical parameters analyzed.

Pooled ORs for NM23 expression, presented in Figure 3 and Table 2, revealed that low NM23 protein levels correlated with N stage (OR = 0.493, 95% CI = 0.269, *P* = 0.904, Figure 3(a)), TNM stage (OR = 0.47, 95% CI = 0.32–0.70, *P* < 0.001, Figure 3(b)), and histological grade (OR = 0.476, 95% CI = 0.32–0.71, *P* = 0.055Figure 3(c)). However, no clear correlation was detected between NM23 expression and T stage (OR = 0.889, 95% CI = 0.50–1.583, *P* = 0.69, Figure 3(d)), lymphatic invasion (OR = 0.801, 95% CI = 0.343–1.874, *P* = 0.609, Figure 3(e)), and vascular invasion (OR = 0.902, 95% CI = 0.429–1.899, *P* = 0.787, Figure 3(f)).

**Figure 4:**
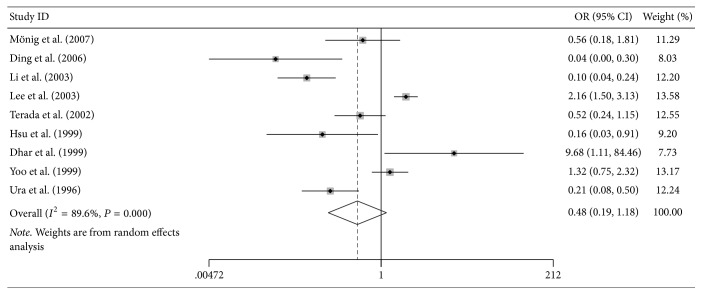
Association between NM23 expression and 5-year OS rate.

There were 9 cohorts with 1592 GC patients evaluating NM23 expression with 5-year OS rate. There was no correlation between NM23 expression and 5-year OS rate (OR = 0.478, 95% CI = 0.194–1.181, *P* = 0.11) (Figure 4).

### 3.4. Sensitivity Analysis and Publication Bias

Sensitivity analysis was conducted through the sequential omission of individual studies. Except that for 5-year OS rate for the NM23 analysis, no single study could change the results, demonstrating that the results of our meta-analysis were quite credible. Moreover, the Egger regression test confirmed the absence of publication bias (Table 2).

## 4. Discussion

NM23 is the first discovered metastasis suppressor gene, which does not influence primary tumor growth but is a powerful inhibitor of metastatic spread of tumors [11]. NM23 is a nucleoside diphosphate kinase, which is thought to be critical for maintenance of intracellular nucleotide homeostasis as a housekeeping function [42]. NM23 also has other enzymatic activities such as histidine kinase, transcriptional activation, and exonuclease activities [43]. NM23 plays a critical role in cell differentiation, adhesion, apoptosis, migration, polymerization, signal transduction pathway, and vascular invasion [44]. Altered NM23 expression was found to be closely related to various tumor metastases, including GC. Several previous reports showed that reduced expression of NM23 correlates with tumor pathology and GC disease prognosis. Chen et al. and Hsu et al. found that the NM23 expression was significantly related to lymph node metastasis [5, 6]. However, it remains controversial despite the numerous independent studies.

To the best of our knowledge, this is the first comprehensive meta-analysis exploring the role of NM23 in GC patients. Our study illustrated that reduced NM23 expression was correlated with elevated gastric cancer risk. And our results suggest that low NM23 levels correlated with higher N stage, worse TNM stage, and poor tumor differentiation grade. However, NM23 levels was not correlated with T stage, lymphatic invasion, vascular invasion, and 5-year OS rate. Overall, it was difficult to firmly establish that the reduced NM23 expression represents a prognostic biomarker for gastric cancer. This would require a larger data set from well-designed studies.

There was no correlation between NM23 expression and 5-year OS rate when we included Lee et al. study. After eliminating the Lee et al. study, the 5-year rate of GC was significantly lower in gastric cancer patients in reduced NM23 expression group than patients with elevated NM23 expression. As Lee et al. study had large sample size and heterogeneity still existed when eliminating the Lee et al. study, it is difficult to make a definite conclusion on the prognostic value of NM23 expression among GC patients. Larger sample size studies from multicenter are needed to further explore this issue.

Although our study revealed the reduced NM23 expression correlated with GC risk, NM23 on predicting prognosis and clinicopathological parameters in GC patients need to be deliberately interpreted. Briefly, the sample size of the included studies was relatively small. Moreover, the criterion of positive NM23 expression was not uniformly defined. Furthermore, we estimated the 5-year OS from Kaplan–Meier curves in some original study, which might be less reliable than the data given by the original paper. What is more, large heterogeneity still remained in some analysis. Finally, owing to only including English and Chinese articles, there might be language bias in some ways. Additionally, positive reports are inclined to be published, which might make certain bias.

## 5. Conclusion

Our meta-analysis indicated that NM23 expression may generally be associated with the gastric cancer risk, but we were unable to determine if NM23 is a potential marker on predicting prognosis and clinicopathological parameters in GC patients. Large-scale and well-designed studies, which use uniform antibody and criterion of NM23 positive expression, are therefore required to further validate the role of the NM23 in predicting gastric cancer progression.

## Figures and Tables

**Table 1:** Main characteristics of the eligible studies.

Author	Year	Country	Cohort	Stage	Methods	5-year OS rate	
						NM23^+^	NM23^−^
Wang	2016	China	230	NA	IHC	NA	
Radovic	2013	Croatia	56	I–IV	IHC	NA	
Okayama	2009	Japan	135	I–III	IHC	NA	
Yang	2008	China	40	I–IV	IHC	NA	
Mönig	2007	Germany	116	I–IV	IHC	35.80%	40.00%
Guan-Zhen	2007	China	71	I–IV	IHC	NA	
Ding	2006	China	78	I–IV	ISH	53.70%	4.17%
Yu	2006	China	74	NA	IHC	NA	
Liu	2005	China	40	II/III	ISH	NA	
Chen	2005	China	43	I–IV	IHC	NA	
Chen	2004	China	43	I–IV	IHC	NA	
Li	2003	China	110	I–IV	IHC	70.50%	20.00%
Lee	2003	Korea	841	I–IV	IHC	64.50%	79.70%
Terada	2002	Japan	103	NA	IHC	63.26%	48.44%
Ji	2002	China	71	I–IV	IHC	NA	
Wang	1999	China	97	NA	IHC	NA	
Hsu	1999	China	24	III	IHC	31.57%	6.90%
Dhar	1999	Japan	59	I–IV	IHC	11.96%	54.06%
Yoo	1999	Korea	261	II-III	IHC	59.82%	53.70%
Wang	1998	China	37	NA	IHC	NA	
Yeung	1998	Australia	23	NA	IHC	NA	
Muller	1998	Germany	529	NA	IHC	NA	
Wei	1997	China	138	I–IV	IHC	NA	
Songun	1996	Holland	105	I–IV	IHC	NA	
Ura	1996	Japan	110	NA	IHC	77.50%	41.50%
Kim	1995	Korea	101	NA	IHC	NA	
Zhang	1995	China	88	I–IV	IHC	NA	

NA, not available; OS, overall survival; IHC, immunohistochemistry; ISH, in situ hybridization.

**Table 2:** Overall analysis of CD133 expression association with clinical features.

	OR	95% CI	*P*	*I* ^2^	*P*_bias_
Gastric cancer risk	0.168	0.063–0.443	<0.001	50.20%	0.134
N stage	0.493	0.269–0.904	0.022	86.10%	0.052
TNM stage	0.47	0.32–0.70	<0.001	82.20%	0.401
Histological grade	0.476	0.319–0.711	0.001	60.30%	0.294
T stage	0.889	0.500–1.583	0.690	69.10%	0.606
Lymphatic invasion	0.801	0.343–1.874	0.609	73.00%	0.437
Vascular invasion	0.902	0.429–1.899	0.787	58.80%	0.835
5-Year OS rate	0.478	0.194–1.181	0.110	89.60%	0.074

*P*_bias_, the *P* value of Egger linear regression test for evaluating publication bias.
